# The importance of increased awareness for delirium in elderly patients with rib fractures after blunt chest wall trauma: a retrospective cohort study on risk factors and outcomes

**DOI:** 10.1186/s12873-019-0248-z

**Published:** 2019-06-13

**Authors:** Ties L. Janssen, Elmand Hosseinzoi, Dagmar I. Vos, Eelco J. Veen, Paul G. H. Mulder, Adrianus M. van der Holst, Lijckle van der Laan

**Affiliations:** 1 Department of surgery, Amphia Hospital Breda, P.O. Box 90518, 4800 RK Breda, The Netherlands; 2 Amphia Academy, Amphia Hospital Breda, Breda, The Netherlands

**Keywords:** Rib fractures, Geriatric trauma, Elderly, Risk factors, Delirium, Adverse outcomes

## Abstract

**Background:**

Rib fractures are common in ageing people after trauma and delirium is a complication often seen in acutely hospitalized elderly patients. For both conditions, elderly have an increased risk for institutionalization, morbidity, and mortality. This study is the first to investigate risk factors of delirium in elderly patients with rib fractures after trauma.

**Methods:**

A retrospective chart review was performed on patients ≥65 years admitted with rib fractures after blunt chest wall trauma to the Amphia hospital Breda, the Netherlands, between July 2013 and June 2018. Baseline patient, trauma- and treatment-related characteristics were identified. The main objectives were identification of risk factors of delirium and investigation of the effect of delirium on outcomes after rib fractures. Outcomes were additional complications, length of hospital stay, need for institutionalization and mortality within six months.

**Results:**

Forty-seven (24.6%) of 191 patients developed a delirium. Independent risk factors for delirium were increased age, physical impairment (lower KATZ-ADL score), nutritional impairment (higher SNAQ score) and the need for a urinary catheter, with odds ratios of 1.07, 0.78, 1.53 and 8.53 respectively. Overall, more complications were observed in patients with delirium. Median ICU and hospital length of stay were 4 and 7 days respectively, of which the latter was significantly longer for delirious patients (*p* < 0.001). Significantly more patients with delirium were discharged to a nursing home or rehabilitation institution (*p* < 0.001). The 6-month mortality in delirious patients was nearly twice as high as in non-delirious patients; however, differences did not reach statistical significance.

**Conclusion:**

Delirium in elderly patients with rib fractures is a serious and common complication, with a longer hospital stay and a higher risk of institutionalization as a consequence. Increased awareness for delirium is imperative, most importantly in older patients, in physically or nutritionally impaired patients and in patients in need of a urinary catheter.

## Introduction

Rib fractures are common fragility fractures in the elderly, frequently present after blunt chest wall trauma [1, 2]. Incidence rates can be underestimated, since these fractures are often missed on initial conventional chest X-rays [3]. Initially, the impact of rib fractures after low energy trauma may also be underestimated in the elderly patients, which can lead to possible disastrous consequences [1]. An increased risk of thoracic morbidity and a mortality rate of up to 22% in elderly patients has previously been described, twice as high as in younger patient [1]. Risk factors for adverse outcomes after rib fractures have been extensively investigated in the elderly. Increased age, a higher number of rib fractures, cardiopulmonary comorbidities, a higher injury severity score (ISS) and the need for a chest tube are risk factors for developing pneumonia, ICU admission, mechanical ventilation, longer hospital stay and death [1, 2, 4–7]. Subsequently, pneumonia in itself is a risk factor for mortality [2]. After sustaining rib fractures, hospitalization is often required due to severity of the associated injuries and the need for adequate pain management.

Delirium is a frequent complication in elderly hospitalized patients. Former studies concluded that delirium is independently associated with increased length of hospital stay, extended mechanical ventilation time, functional decline, increased health-care costs and death within one year [8, 9]. Acute pain, respiratory distress and acute hospitalization are known risk factors for delirium and are, also, three important features that are frequently present in patients with rib fractures [8, 10, 11].

No previously published research has investigated risk factors and outcomes of delirium in elderly patients with rib fractures. Focus seems to lay on delirium in cardiac patients and patients with hip fractures, since high incidences of delirium are seen in these patient groups [12, 13]. However, our population is aging and the global population of people over 65 years of age has grown from 5% in 1960 to over 8.5% in 2017 [14]. The amount of fragility fractures, and with that rib fractures, is expected to increase due to this aging population [15]. Since higher age is a risk factor for developing delirium [8], it is important to assess delirium incidence and its consequences in this specific population.

The main objective of this study is to describe the incidence, risk factors and outcomes of delirium in elderly patients who have been acutely hospitalized with rib fractures due to blunt chest wall trauma.

## Methods

### Study design and participants

A retrospective cohort study was conducted including all patients aged ≥65 years with one or more rib fractures after blunt trauma, admitted two a level II trauma centre and tertiary teaching hospital in Breda, the Netherlands, between July 2013 and June 2018. The Amphia Hospital is centred in a large crowded region and the hospital covers about 400.000 inhabitants. Each year 20.000 patients visit the emergency department for surgically related diseases; more than half of them are trauma patients. Over 1500 trauma patients are admitted each year and treated for their fractures. Data were retrieved through retrospective chart review, using a full electronic patient file system: Hyperspace Version IU4 (Epic, Inc., Verona, WI).

### Baseline characteristics

Baseline patient characteristics (age, gender, BMI, intoxications, sensory impairment and comorbidities) were assessed. The severity of comorbidity was determined by the Charlson Comorbidity Index (CCI) [16]. Data on previous rib fractures, history of delirium, cognitive impairment, cardiac, pulmonary or neurological comorbidities, renal insufficiency, diabetes or osteoporosis in medical history, hypertension, hypercholesterolemia, and a malignancy in history were registered. Nutritional status and physical independence were routinely scored by ward nurses at admission to the wards, using the Short Nutritional Assessment Questionnaire (SNAQ) and the KATZ-Activities of Daily Living (KATZ-ADL) score respectively. A lower SNAQ score represents a low risk of undernourishment, while a SNAQ score of ≥2 represents malnourishment [17]. The KATZ-ADL score is a score from 0 to 6, of which 6 represents physical independence and a score of 5 or lower indicates physical impairment [18, 19].

### Trauma- and treatment-related characteristics

The mechanism of injury, the number and laterality of rib fractures, additional intrathoracic injuries and injury severity were assessed. The mechanism of injury was divided in low energetic trauma (LET) and high energetic trauma (HET), according to the ninth edition of ATLS [20]. The severity of the trauma was scored according to the ISS [21].

Conventional chest X-rays and thoracic CT-scans were used to assess the number and location of the rib fractures, to diagnose a pneumothorax, haemothorax, lung contusion or a flail chest and to assess the need for a chest tube. Thoracic CT-scans were routinely available, however were performed only when conventional chest X-rays were inconclusive. Imaging reports from the radiologist at the time of admission were used and images were not reassessed. Surgical rib fixation was indicated in case of a flail chest or persistent excessive pain despite maximum pain management.

When patients were admitted to the surgical ward, treatment consisted of adequate respiratory support, sufficient pain management, chest physical therapy, consultation of a pulmonologist or geriatrician when indicated, active mobilisation and nasal oxygen supplementation. Pain management and therapy were based on the Visual Analogue Score (VAS) [22] and analgesia was given according to the WHO analgesics ladder. The use of prophylactic or therapeutic antipsychotics was started according to hospital protocol. Cognitively impaired patients, patients with delirium in history and frail patients were given prophylactic haloperidol. A geriatrician was consulted to confirm diagnosis when delirium was suspected and started therapeutic haloperidol. If necessary, a physician could choose to deviate from the protocol.

Unplanned ICU admission, length of ICU stay, length of hospital stay (LOS) and the need for intubation were registered. Patients were intubated in case of respiratory failure with inadequate oxygenation and ventilation.

### Objectives

The main study objectives were to describe the incidence of delirium, to identify risk factors associated with delirium and to investigate the effect of delirium on secondary outcomes after rib fractures in elderly patients admitted to the hospital after blunt chest wall trauma.

### Outcomes

The primary outcome was incidence of delirium. Delirium was screened using the Delirium Observation Screening Score (DOSS) [23], which was scored three times a day by a nurse while providing regular care. Delirium was suspected when DOSS was three or more, in which case a geriatrician was consulted. The geriatrician confirmed the diagnosis using the DSM-IV criteria or the Confusion Assessment Method [24, 25].

The secondary outcomes were additional adverse events next to delirium, LOS, length of ICU stay, institutionalization after discharge, the need for home care after discharge, and mortality during admission, after one month and after six months. Any deviation from the normal postoperative course was considered an additional adverse event. The CompeT&T (Eindhoven) database was used to assess (date of) mortality.

### Statistical analysis

All baseline characteristics and trauma- and treatment-related variables were considered risk factors for developing delirium and were compared between patients who developed delirium and those who did not. Dichotomous variables were presented as (relative) frequencies and were compared between the two groups using the Pearson chi-squared test or Fisher’s Exact test. Continuous variables with a skewed distribution were summarized as medians with interquartile ranges [IQR] and compared between the two groups using the Mann-Whitney test. Variables with a univariable *p*-value below 0.30 were selected to simultaneously enter a multivariable logistic regression model with delirium as outcome. In that model a stepwise backward elimination method was used to further delete variables one by one from the model based on the largest *p*-value above 0.30. This selection process was done separately for each of the two sets of independent variables mentioned above in the methods section. Next, both selected subsets of remaining variables were combined to enter a new logistic regression model, again using a stepwise elimination method to further delete variables based on the largest *p*-value above 0.30. Effects of the explanatory variables on delirium as estimated in the final model were expressed by means of odds ratios with 95% confidence intervals and *p*-values. An effect was considered significant if its p-value dropped below 0.05.

Statistical analysis was performed using SPSS Version 23.0 (SPSS Inc., Chicago, Illinois, USA) software. Missing data were not imputed.

This manuscript was composed using the checklist provided in the STROBE Statement for cohort studies [26].

## Results

Between July 2013 and June 2018, 191 patients over 65 years of age with one or more rib fractures after blunt chest wall trauma were admitted. Delirium occurred in 47 (24.6%) patients, with a median duration of 7 days (IQR 4–12). Patients that developed a delirium were older, were undernourished or physically impaired more often and had a greater burden of comorbidity compared to people that did not develop a delirium. A complete overview of baseline characteristics, including separate display of delirious and non-delirious patients, is shown in Table 1.

**Table 1:** Baseline characteristics of elderly patients with rib fractures

	Total *N* = 191 (%)	Patients with delirium *N* = 47 (%)	Patients without delirium *N* = 144 (%)	*p*-value
Age (median in years, IQR)	78 (72–86)	83 (77–89)	77 (71–85)	0.004
Gender (male, %)	107 (56.0)	28 (59.6)	79 (54.9)	0.57
Body Mass Index (median, IQR)^a^	25.3 (23.1–29.2)	25.5 (21.9–29.1)	25.2 (23.3–29.2)	0.58
Comorbidities				
Charlson Comorbidity Index (median, IQR)	5 (3–6)	5 (4–6)	4 (3–6)	0.020
Previous rib fractures	6 (3.1)	1 (2.1)	5 (3.5)	1.00
History of delirium	23 (12.0)	10 (21.3)	13 (9.0)	0.025
Cognitive impairment	23 (12.0)	8 (17.0)	15 (10.4)	0.23
Cardiac	75 (39.3)	20 (42.6)	55 (38.2)	0.60
Pulmonary	48 (25.1)	12 (25.5)	36 (25.0)	0.94
Neurologic	58 (30.4)	18 (38.3)	40 (27.8)	0.17
Renal	17 (8.9)	6 (12.8)	11 (7.6)	0.37
Osteoporosis	14 (7.3)	2 (4.3)	12 (8.3)	0.52
Diabetes	39 (20.4)	10 (21.3)	29 (20.1)	0.87
Hypertension	77 (40.3)	21 (44.7)	56 (38.9)	0.48
Hypercholesterolaemia	12 (6.3)	2 (4.3)	10 (6.9)	0.73
Malignancy	39 (20.4)	11 (23.4)	28 (19.4)	0.56
Sensory impairment				
Hearing impairment	61 (31.9)	17 (36.2)	44 (30.6)	0.47
Visual impairment	63 (33.0)	19 (40.4)	44 (30.6)	0.21
Nutritional status				
SNAQ – RC (median, IQR)^a^	0 (0–0)	0 (0–1.25)	0 (0–0)	0.016
Undernourishment (SNAQ-RC ≥ 2)	24 (13.0)	11 (23.9)	13 (9.4)	0.011
Physical status				
KATZ-ADL score (median, IQR)^a^	6 (4–6)	5 (3–6)	6 (5–6)	0.007
Physical impairment (KATZ-ADL < 6)	71 (38.4)	25 (53.2)	46 (33.3)	0.016
Intoxications				
Alcohol consumption^b^	80 (55.9)	21 (56.8)	59 (55.7)	0.91
Active smoker^b^	21 (14.6)	7 (19.4)	14 (13.0)	0.34
History of smoking^b^	87 (64.4)	25 (71.4)	62 (62.0)	0.32

^a^ Missing data < 5%; ^b^ Missing data 25–30%

The variables shown in Table 1 with a *p*-value below 0.30 were selected to simultaneously enter the first logistic regression model. In the logistic regression analysis, the following variables were successively deleted from the model: cognitive impairment, visual impairment, Charlson Comorbidity Index, and neurologic comorbidity.

Table 2 provides an overview of the trauma mechanisms, diagnostics, amount and laterality of rib fractures and additional intrathoracic injuries, and initial treatment. The most common additional injuries were pneumothorax and haemothorax, for which 28 patients received a chest tube. In over 50% of patients, treatment with oral analgesics was not sufficient. They were therefore given either epidural analgesia or analgesia via PCA pump.

**Table 2:** Characteristics of trauma, injuries and interventions during admission

	Total *N* = 191 (%)	Patients with delirium *N* = 47 (%)	Patients without delirium N = 144 (%)	*p*-value
Energy of trauma				
Low	142 (74.3)	34 (72.3)	108 (75.0)	0.72
High	49 (25.7)	13 (27.7)	36 (25.0)	
Injury Severity Score				
ISS < 16	157 (82.2)	36 (76.6)	121 (84.0)	0.25
ISS ≥ 16	34 (17.8)	11 (23.4)	23 (16.0)	
Rib fractures				
1–2	62 (32.5)	16 (34.0)	46 (31.9)	0.89
3–4	78 (40.8)	18 (38.3)	60 (41.7)	
≥ 5	53 (27.7)	14 (29.8)	39 (27.1)	
Total, median (IQR)	3 (2–5)	3 (2–5)	3 (2–5)	0.84
Laterality of rib fractures				
Unilateral	184 (96.3)	44 (93.6)	140 (97.2)	0.37
Bilateral	7 (3.7)	3 (6.4)	4 (2.8)	
Additional intrathoracic injuries				
Haemothorax	27 (14.1)	10 (21.3)	17 (11.8)	0.11
Pneumothorax	34 (17.8)	13 (27.7)	21 (14.6)	0.042
Flail chest	9 (4.7)	5 (10.6)	4 (2.8)	0.042
Lung contusion	16 (8.4)	5 (10.6)	11 (7.6)	0.55
VAS and analgesics				
VAS at admission, median (IQR)^a^	5 (3.25–6)	4 (3–6)	5 (4–6.5)	0.14
Oral paracetamol	188 (98.4)	47 (100)	141 (97.9)	1.00
Oral paracetamol + opioids	162 (84.8%)	37 (78.7)	130 (90.3)	0.038
PCA pump	52 (27.2)	18 (38.3)	34 (23.6)	0.050
Epidural	45 (23.6)	15 (31.9)	30 (20.8)	0.12
Interventions				
Urinary catheter	75 (39.3)	34 (72.3)	41 (28.5)	< 0.001
Mechanical ventilation	12 (6.3)	6 (12.8)	6 (4.2)	0.075
Chest tube	28 (14.7)	11 (23.4)	17 (11.8)	0.051
Lung physiotherapist	162 (84.8)	39 (83.0)	123 (85.4)	0.69
Consultation of pulmonologist	50 (26.2)	21 (44.7)	29 (20.1)	0.001
Consultation of geriatrician	85 (44.5)	42 (89.4)	43 (29.9)	< 0.001
Unplanned ICU admission	34 (17.8)	18 (38.3)	16 (11.1)	< 0.001
Surgery	10 (5.2)	5 (10.6)	5 (3.5)	0.068
Median ASA score	3			
Operation time (minutes, min –max)	49–231			
Blood loss (cc, min –max)	10–1000			
Preoperative anaemic patients	5 (50)			
Postoperative anaemic patients	8 (80)			

^a^Missing data <10%

A total of thirty-four patients were admitted to the ICU, of which twelve patients needed to be intubated due to the severity of their injuries. Incidence of delirium was significantly higher (p < 0.001) in these ICU patients. Ten patients had to undergo rib fixation surgery, either to treat a flail chest or to relieve severe pain caused by the extent of their injuries. Fifty percent of these surgical patients developed a delirium during their admission.

Similar to the first model, the variables shown in Table 2 with a p-value below 0.30 were selected to simultaneously enter the second logistic regression model. In the logistic regression analysis, the following variables were successively deleted from the model: endotracheal intubation, the need for surgery, presence of a haemothorax, PCA pump use, epidural analgesia, ISS ≥16, and presence of a pneumothorax.

The two sets of variables remaining after backward elimination in the first two models were combined to enter a final logistic regression model. By looking for interactions between the explanatory variables we found an interaction between Age and KATZ-ADL, which should be integrated in our model. Backward elimination based on the highest *p*-value above 0.30 resulted in a deletion of VAS score, the need of a chest drain, presence of a flail chest and oral opioid use.

The variables age and KATZ-ADL were centered around their respective means of 79.05 years and 4.96 points, so that the main effect of age holds for average KATZ-ADL and the main effect of KATZ-ADL holds for average age. With increasing age the effect of KATZ-ADL decreases; the *p*-value of this effect modification was 0.059, which was considered relevant to take into account. The total number of missing observations was 7 out of 191, of which 1 out of 47 delirium cases. The *p*-value of the significance of the model was *p* < 0.001.

The results of our final logistic regression model are presented in Table 3. Increased age, lower KATZ-ADL score, a higher SNAQ score and the need of a urinary catheter are independent risk factors for delirium. For an average KATZ-ADL score, the odds ratio of age was 1.07 (95% CI; 1.01–1.13; *p* = 0.030). The odds ratio of KATZ-ADL was 0.78 (95% CI; 0.61–1.00; *p* = 0.048), which effect appeared to decrease at higher than average ages. Odds ratios of SNAQ score and the need for a urinary catheter were 1.53 (95% CI; 1.09–2.16; *p* = 0.015) and 8.53 (95% CI; 3.21–22.7; *p* < 0.001) respectively.

**Table 3:** Multivariable analysis for independent risk factors for delirium after rib fractures

	Coefficient	SE	Odds ratio (95% Confidence interval)	*p*-value
Age	0.0639	0.0294	1.07 (1.01–1.13)	0.030
KATZ – ADL	−0.2462	0.1242	0.78 (0.61–1.00)	0.048
SNAQ – RC	0.4272	0.1760	1.53 (1.09–2.16)	0.015
History of delirium	0.8688	0.5663	2.38 (0.79–7.23)	0.12
Unplanned ICU admission	0.8278	0.5158	2.29 (0.83–6.29)	0.11
Urinary catheter	2.1440	0.4991	8.53 (3.21–22.7)	< 0.001
Age x KATZ-ADL interaction	0.0276	0.0146	1.03 (1.00–1.06)	0.059

Significance of model: chi-square = 58.661; df = 7; *p* < 0.001

Table 4 shows the outcomes after rib fractures and their association with delirium. A significantly longer hospital stay was seen in patients that developed delirium (7 days, IQR 4–12 versus 6 days, IQR 8–21; *p* < 0.001). Patients with delirium during admission were significantly more in need of extra care after discharge, either at home or in an institution (*p* < 0.001 for both outcomes). Over 10 % of all patients died within the first six months. Mortality rates after 1 month and 6 months were roughly twice as high in patients with delirium, although this difference did not reach a statistical significance.

**Table 4:** Outcomes after rib fractures in patients with and without delirium

	Total N = 191 (%)	Patients with delirium N = 47 (%)	Patients without delirium N = 144 (%)	*p*-value
Complications				
Any complication other than delirium	51 (26.7)	19 (40.4)	32 (22.2)	0.014
Pneumonia	40 (20.9)	12 (25.5)	28 (19.4)	0.37
Length of ICU and hospital stay				
Length of ICU stay in days, median (IQR)	4 (1.0–6.0)	4.5 (1.0–6.0)	3.5 (1.25–6.0)	0.93
Length of hospital stay, median (IQR)	7 (4–12)	15 (8.0–21.0)	6 (3.0–9.75)	< 0.001
Discharge location				
New location	61 (31.9)	28 (59.6)	33 (22.9)	< 0.001
Same location	121 (63.4)	16 (34.0)	105 (72.9)	
Home care				
Discharge home with care	52 (27.2)	14 (29.8)	38 (26.4)	< 0.001
Discharge home without care	69 (36.1)	2 (4.3)	67 (46.5)	
Mortality				
During admission	9 (4.7)	3 (6.4)	6 (4.2)	0.69
1 month	12 (6.3)	5 (10.6)	7 (4.9)	0.17
6 months	21 (11.0)	8 (17.0)	13 (9.0)	0.13

## Discussion

Delirium is an often-described topic in elderly patients and has considerable adverse outcomes with a great healthcare burden. This study aimed to describe the incidence of delirium in elderly patients with rib fractures, to describe the associated outcomes of these rib fractures and to identify independent risk factors for developing a delirium during admission.

Delirium occurred in nearly 25 % of patients in this study, which is similar to reports in earlier studies. In a 2018 study by O’Connell et al. investigating the effects of different types of analgesia on the incidence of delirium in elderly patients with rib fractures, 31.9% developed a delirium [27]. Two additional studies reported incidences of delirium of 23.3 and 36.7% in trauma patients acutely admitted to the hospital, however they did not specifically investigate this outcome in elderly patients with rib fractures [28, 29].

In line with previous studies, age, a lower KATZ-ADL score and the presence of a urinary catheter were found to be independent risk factors for delirium in elderly patients with rib fractures [8, 11, 28, 30]. Cognitive impairment, a history of delirium, functional impairment, sensory impairment and severity of comorbidities have also been shown to be predisposing risk factors for delirium in elderly surgical patients in general [8]. These risk factors could not be confirmed for elderly patients with rib fractures by the current research.

Patients with a poor nutritional status have a higher risk for rib fractures due to diminished bone mineral density [31]. Additionally, previous studies in patients with hip fractures showed malnutrition to be an independent risk factor for delirium [32]. By combining these findings, evidence suggests that a poor nutritional status not only makes you susceptible to rib fractures after trauma, but also for delirium after incurring these rib fractures. The current research together with another recent study, supports these findings by concluding that a SNAQ score ≥ 3 increases the risk for delirium [33].

Overall, significantly more complications were observed in patients with delirium. A similar effect could not be observed for pneumonia. A previous study in patients undergoing elective total hip arthroplasty showed that the risk of delirium is increased when patients develop pneumonia or myocardial infarction as complications, with odds ratios of 2.0 and 3.8 respectively [34]. In contrast, another study did not show pneumonia to be a risk factor for delirium in osteoporotic hip fracture patients, which is a population more similar to ours [30]. Effective pain management diminishes pulmonary complications and decreases the risk for delirium thanks to increased lung capacity, by which a patient can breathe unburdened. Additionally, pain management can also effectively prevent other complications that may lead to a delirium [35].

Admission to the ICU is often required to ensure adequate respiratory function in elderly patients with rib fractures. Bryczkowski et al. stated that for every year older than 50, chance of delirium increases by 10% on the surgical ICU [36]. After logistic regression analysis, unplanned ICU admission could not be identified as an independent risk factor for delirium, even though significantly more patients in our population that were admitted to the ICU developed a delirium. Using a bigger sample might shift the influence of ICU admission on delirium to a significant result, however additional prospective studies should be conducted in the future to prove this claim.

Previous studies investigating outcomes after delirium showed a significantly prolonged LOS and an increased risk of institutionalization in delirious patients [28, 29, 37, 38]. In line with these studies, the current research showed LOS to be significantly prolonged in delirious patients, with a LOS more than two times as long as in patients without a delirium. Delirious patients were significantly more in need of additional home care, physical support or extended recovery time in a nursing home or revalidation centre.

Six-month-mortality after rib fractures was nearly twice as high in patients that developed a delirium during admission, a finding considered by the authors to be relevant, even though this difference was not statistically significant. A recent meta-analysis concluded that delirium during admission significantly increases risk of mortality after a median follow-up period of 22.7 months (hazard ratio 1.95) [38]. Also, higher in-hospital mortality rates were seen for delirious patients in another recent study [29]. The relatively short follow-up period and the number of events are possible causes for differences in results in our study compared to these other studies. Prolonged follow-up and a bigger sample are therefore required.

Fifty percent of the patients that underwent rib fracture fixation in this study developed delirium during admission, without significant differences between groups. This lack of significance is likely explained by the small number of patients undergoing surgery, since opposite to the treatment of other trauma-related fractures such as hip fractures, the key components of the treatment of rib fractures is adequate pain management and respiratory support [39–41]. Still, increased attentiveness for delirium after rib fixation surgery is recommended.

### Limitations

The design of this study and its retrospective nature made the risk of bias relatively high. Due to its design, considerable amounts of data were missing.

Although routinely available, hospital protocols do not require a CT thorax to be made for every trauma patient. The number of rib fractures and additional injuries in most patients were diagnosed using a conventional x-ray. Previous research has shown that the number of rib fractures can be underestimated or rib fractures can even be missed when only using a chest x-ray for diagnosis [3, 42].

The DOSS was used to screen for delirium by ward nurses. Recent validation studies have shown inconclusive results, with sensitivity rates varying from 56 to 91% [23, 43]. This difference is likely explained due to difficulty in diagnosing the hypoactive subtype of delirium. This subtype is present in over 40% of cases and is unrecognized in over half of these cases [44].

### Recommendations

When acutely admitting an elderly patient with rib fractures due to blunt chest wall trauma, increased awareness for delirium is recommended. This awareness, together with identification of patients most at risk and primary preventive measures such as those stated in the HELP guidelines [45], might be able to lower the incidence of delirium. When admitting elderly patients with rib fractures, adequate pain management, respiratory support and chest physiotherapy [41], together with early consultation of a geriatrician at admission, are advised.

In the future, more extensive prospective research should be conducted to identify adequate methods to prevent delirium in elderly patients with fractured ribs. Larger samples are needed to determine specific risk factors for delirium in this population. With these risk factors, patients most at risk for developing delirium and patients most in need of primary prevention can be identified.

## Conclusion

Delirium is a serious and common complication, occurring in nearly a quarter of elderly patients with rib fractures after traumatic chest wall injury. Patients with rib fractures who develop delirium have an increased risk of a prolonged hospital stay and have an increased risk of institutionalization. Increased awareness for delirium in these patients is imperative, most importantly in older patients, patients with a lower KATZ-ADL score, patients with a higher SNAQ score and those in need of a urinary catheter.

## Data Availability

The datasets generated and/or analysed during the current study are not made publicly available due to privacy reasons.

## Abbreviations

ASA: American Society of Anaesthesiologists
ATLS: Advanced Trauma Life Support
BMI: Body Mass Index
CCI: Charlson Comorbidity Index
DOSS: Delirium Observation Screening Score
DSM-IV: Diagnostic and Statistical Manual of Mental Disorders
HELP: Hospital Elder Life Program
HET: High energetic trauma
ICU: Intensive Care Unit
IQR: Interquartile Range
ISS: Injury Severity Score
KATZ-ADL: KATZ – Activities of Daily Living
LET: Low energetic trauma
LOS: Length of hospital Stay
PCA: Patient Controlled Analgesia
SNAQ: Short Nutritional Assessment Questionnaire
VAS: Visual Analogue Score
WHO: World Health Organisation

## References

[1] Bulger EM, Arneson MA, Mock CN (2000). Rib fractures in the elderly. J Trauma.

[2] Battle CE, Hutchings H, Evans PA (2012). Risk factors that predict mortality in patients with blunt chest wall trauma: a systematic review and meta-analysis. Injury..

[3] Sano A. Rib radiography versus chest computed tomography in the diagnosis of rib fractures. Thorac Cardiovasc Surg. 2018. 10.1055/s-0038-1645887 29715707

[4] Shulzhenko NO, Zens TJ, Beems MV (2017). Number of rib fractures thresholds independently predict worse outcomes in older patients with blunt trauma. Surgery..

[5] Van Vledder MG, Kwakernaak V, Hagenaars T, et al. Patterns of injury and outcomes in the elderly patient with rib fractures: a multicenter observational study. Eur J Trauma Emerg Surg. 2018. 10.1007/s00068-018-0969-9 PMC6689021 29905897

[6] Barry R, Thompson E (2018). Outcomes after rib fractures in geriatric blunt trauma patients. Am J Surg.

[7] Sawa J, Green RS, Thoma B (2018). Risk factors for adverse outcomes in older adults with blunt chest trauma: a systematic review. CJEM..

[8] Inouye SK, Westendorp RG, Saczynski JS (2014). Delirium in elderly people. Lancet..

[9] Leslie DL, Inouye SK (2011). The importance of delirium: economic and societal costs. J Am Geriatr Soc.

[10] Inouye SK, Charpentier PA (1996). Precipitating factors for delirium in hospitalized elderly persons. Predictive model and interrelationship with baseline vulnerability. JAMA..

[11] Marcantonio ER (2017). Delirium in Hospitalized Older Adults. N Engl J Med.

[12] Yang Y, Zhao X, Dong T (2017). Risk factors for postoperative delirium following hip fracture repair in elderly patients: a systematic review and meta-analysis. Aging Clin Exp Res.

[13] Crocker E, Beggs T, Hassan A (2016). Long-term effects of postoperative delirium in patients undergoing cardiac operation: a systematic review. Ann Thorac Surg.

[14] The World Bank. Bank TW. Population ages 65 and above (% of total); 2017. https://data.worldbank.org/indicator/SP.POP.65UP.TO.ZS. Accessed 21 Mar 2019.

[15] Palvanen M, Kannus P, Niemi S (2004). Hospital-treated minimal-trauma rib fractures in elderly Finns: long-term trends and projections for the future. Osteoporos Int.

[16] Charlson ME, Pompei P, Ales KL (1987). A new method of classifying prognostic comorbidity in longitudinal studies: development and validation. J Chronic Dis.

[17] Kruizenga HM, Seidell JC, de Vet HC (2005). Development and validation of a hospital screening tool for malnutrition: the short nutritional assessment questionnaire (SNAQ). Clin Nutr.

[18] Wallace M, Shelkey M (2007). Hartford Institute for Geriatric N. Katz index of Independence in activities of daily living (ADL). Urol Nurs.

[19] Cabanero-Martinez MJ, Cabrero-Garcia J, Richart-Martinez M (2009). The Spanish versions of the Barthel index (BI) and the Katz index (KI) of activities of daily living (ADL): a structured review. Arch Gerontol Geriatr.

[20] Subcommittee A (2013). American College of Surgeons' committee on T, international Awg. Advanced trauma life support (ATLS(R)): the ninth edition. J Trauma Acute Care Surg.

[21] Baker SP, O'Neill B, Haddon W (1974). The injury severity score: a method for describing patients with multiple injuries and evaluating emergency care. J Trauma.

[22] Katz J, Melzack R (1999). Measurement of pain. Surg Clin North Am.

[23] Gavinski K, Carnahan R, Weckmann M (2016). Validation of the delirium observation screening scale in a hospitalized older population. J Hosp Med.

[24] American Psychiatric Association. Diagnostic and statistical manual of mental disorders. 5th ed. Arlington: Author; 2013.

[25] Inouye SK, van Dyck CH, Alessi CA (1990). Clarifying confusion: the confusion assessment method. A new method for detection of delirium. Ann Intern Med.

[26] Vandenbroucke JP, von Elm E, Altman DG (2014). Strengthening the reporting of observational studies in epidemiology (STROBE): explanation and elaboration. Int J Surg.

[27] O'Connell KM, Quistberg DA, Tessler R (2018). Decreased risk of delirium with use of regional analgesia in geriatric trauma patients with multiple rib fractures. Ann Surg.

[28] de Castro SM, Unlu C, Tuynman JB (2014). Incidence and risk factors of delirium in the elderly general surgical patient. Am J Surg.

[29] Schubert M, Schurch R, Boettger S (2018). A hospital-wide evaluation of delirium prevalence and outcomes in acute care patients - a cohort study. BMC Health Serv Res.

[30] Kim JY, Yoo JH, Kim E (2017). Risk factors and clinical outcomes of delirium in osteoporotic hip fractures. J Orthop Surg (Hong Kong).

[31] Urena P, Bernard-Poenaru O, Ostertag A (2003). Bone mineral density, biochemical markers and skeletal fractures in haemodialysis patients. Nephrol Dial Transplant.

[32] Mazzola P, Ward L, Zazzetta S (2017). Association between preoperative malnutrition and postoperative delirium after hip fracture surgery in older adults. J Am Geriatr Soc.

[33] van Eijsden WA, Raats JW, Mulder PG (2015). New aspects of delirium in elderly patients with critical limb ischemia. Clin Interv Aging.

[34] Aziz KT, Best MJ, Naseer Z (2018). The Association of Delirium with perioperative complications in primary elective Total hip arthroplasty. Clin Orthop Surg.

[35] May L, Hillermann C, Patil S (2016). Rib fracture management. BJA Education.

[36] Bryczkowski SB, Lopreiato MC, Yonclas PP (2014). Risk factors for delirium in older trauma patients admitted to the surgical intensive care unit. J Trauma Acute Care Surg.

[37] Gleason LJ, Schmitt EM, Kosar CM (2015). Effect of delirium and other major complications on outcomes after elective surgery in older adults. JAMA Surg.

[38] Witlox J, Eurelings LS, de Jonghe JF (2010). Delirium in elderly patients and the risk of postdischarge mortality, institutionalization, and dementia: a meta-analysis. JAMA..

[39] Wardhan R (2013). Assessment and management of rib fracture pain in geriatric population: an ode to old age. Curr Opin Anaesthesiol.

[40] Galvagno SM, Smith CE, Varon AJ (2016). Pain management for blunt thoracic trauma: a joint practice management guideline from the eastern Association for the Surgery of trauma and trauma anesthesiology society. J Trauma Acute Care Surg.

[41] Kourouche S, Buckley T, Munroe B (2018). Development of a blunt chest injury care bundle: an integrative review. Injury..

[42] CEt M, Raja AS, Baumann BM (2017). Rib fracture diagnosis in the Panscan era. Ann Emerg Med.

[43] Hasemann W, Tolson D, Godwin J (2018). Nurses' recognition of hospitalized older patients with delirium and cognitive impairment using the delirium observation screening scale: a prospective comparison study. J Gerontol Nurs.

[44] Janssen TL, Alberts AR, Hooft L, et al. Prevention of postoperative delirium in elderly patients planned for elective surgery: systematic review and meta-analysis. Clin Interv Aging. 2019; [Accepted]. 10.2147/CIA.S201323 PMC6590846 31354253

[45] Inouye SK, Bogardus ST, Baker DI (2000). The hospital elder life program: a model of care to prevent cognitive and functional decline in older hospitalized patients. Hospital elder life program. J Am Geriatr Soc.

